# Effect of congenital heart disease on the recurrence of cough variant asthma in children

**DOI:** 10.1186/s12872-021-01940-8

**Published:** 2021-03-10

**Authors:** B. W. Feng, C. Y. He, X. Q. Liu, Y. S. Chen, S. R. He

**Affiliations:** 1 The Second School of Clinical Medicine, Southern Medical University, Guangzhou, Guangdong China; 2 Department of Neonatology of Guangdong Provincial People’s Hospital, Guangdong Academy of Medical Sciences, Guangzhou, Guangdong China; 3 Department of Epidemiology, Guangdong Provincial People’s Hospital, Guangzhou, Guangdong China; 4 Department of Pediatrics of Guangdong Provincial People’s Hospital, 106 Zhongshan Er Road, Guangzhou, 510080 Guangdong China

**Keywords:** CHD, CVA, Hazard of recurrent CVA

## Abstract

**Background:**

The research into the recurrence of cough variant asthma (CVA) in congenital heart disease (CHD) are few in number. The purpose of this study is to investigate the effect of CHD on the risk of the recurrence of CVA.

**Methods:**

This study was a retrospective cohort study of 489 children with CVA aged between one and 14 years, of whom 67 had CHD complicated with CVA and 134 had CVA without CHD at a ratio of 1:2 according to age, sex and index year. The adjusted hazard ratio (aHR) of CVA recurrence in both the CHD cohort and the non-CHD cohort was determined by multivariate analysis using the Cox proportional hazard regression model.

**Results:**

Adjusting for CHD classification, Mycoplasma pneumonia (MP) infection and immunoglobulin E (IgE) sensitization, the recurrence hazard of CVA in the complex congenital heart disease (CCHD) group (aHR = 3.281; 95% CI 1.648–6.530; *P* < 0.01) was significantly higher than that in the simple congenital heart disease group (aHR = 2.555; 95% CI 1.739–3.752; *P* < 0.01). Further, children with IgE sensitization (aHR = 2.172; 95% CI 1.482–3.184; *P* < 0.01) had a higher recurrence hazard of CVA than those without IgE sensitization, and children with MP infection (aHR = 1.777; 95% CI 1.188–2.657; *P* < 0.01) had a higher recurrence hazard of CVA than those without the MP infection.

**Conclusion:**

The hazard of recurrent CVA is higher in children with CHD, especially in the CCHD children. In addition, those children with IgE sensitization or a MP infection had an increased hazard of recurrent CVA.

## Introduction

Asthma is a chronic airway inflammatory disease characterized by bronchial hyperresponsiveness and reversible airflow obstruction. A cause of health problems globally for all ages, asthma affects approximately 300 million people worldwide. Its incidence rate, treatment costs and burden on health care systems in many developed nations such as China, Australia, New Zealand, Canada and the United Kingdom, are increasing [1]. The Global Initiative on the Prevention and Treatment of Asthma (GINA) defines cough variant asthma (CVA) as a special type of asthma without wheezing or shortness of breath, where coughing is the sole or main symptom [1]. CVA in children is a particular type of asthma, predominantly characterized by a persistent cough. Children with CVA have no typical clinical symptoms and are easy to misdiagnose, resulting in their being unable to receive standardized treatment. The disease can then develop into typical asthma, hence affecting the child’s growth. CVA in children can be prevented, but it is difficult to cure, and always causes repeated attacks. Therefore, discovering the hazard factors of CVA is of great importance in the prevention and provision of relevant treatment for it.

In contrast, the total number of children diagnosed with congenital heart disease (CHD) has been rising due to improved diagnosis. However, there has been no improvement in its treatment. Over the past 60 years, great progress has been made in the medical and surgical care of these children. However, specialists are facing challenges as these children develop new complications [2]. They often need long-term follow-up for lifelong heart problems and decades of reoperations to carry out surgical repairs that may even lead to various respiratory diseases. For children with CHD complicated by CVA, it is necessary to carry out further studies into the likelihood of incidence and recurrence rates increasing. At the same time, the relationship between the severity of airflow obstruction and cardiovascular disease’s morbidity and mortality has been well established. However, the relationship between CVA characterized by variable airway obstruction and cardiovascular disease is not clear, and data reported on the relationship between asthma and cardiovascular disease have not been confirmed [3].

In addition, considering the gradual increase in the incidence and recurrence rates of CVA, children with CHD are of great interest. The effect of CHD on the recurrence rate of CVA in children has rarely been studied till now.

## Methods

**Figure 1:**
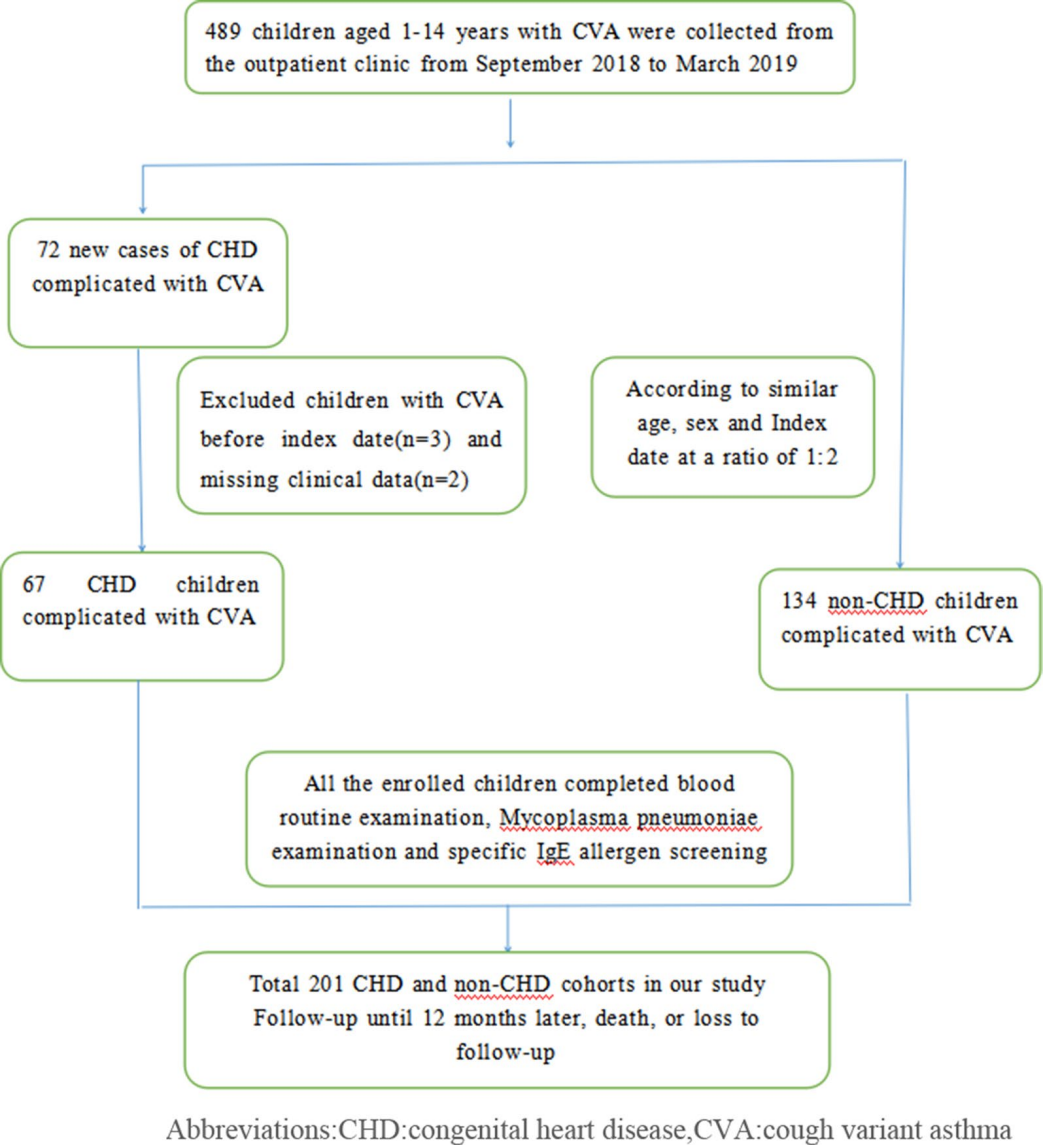
Flow chart presenting the selection of study children

A total of 489 children aged between one and 14 years with CVA were recruited from the hospital-based outpatient Respiratory Disease Clinic during the period from September 2018 to March 2019. The diagnostic criteria of CVA were based on the Guidelines for the Diagnosis and Prevention of Bronchial Asthma in Children, 2016 [4]. Figure 1 depicts the cohort selection process. We identified 67 cases of CHD complicated by CVA from their medical history and color doppler ultrasound examination from these children. The date of the CVA diagnosis was set as the index date. Index date is the time when the children were enrolled, and the starting time of each group of experimental studies. The starting time of each group of patients may be different. We then frequency matched the 134 children with CVA but without CHD, in a ratio of 1:2 according to sex, age and index date. The Medical Ethics Committee of Guangdong Provincial People's Hospital reviewed the project and obtained the written informed consent of the parents or guardians. All the children were given routine blood tests, Mycoplasma pneumoniae antibodies and allergen specific IgE tests, and their family history was collated. After their clinical symptoms had been controlled, they were followed up for 12 months. During the follow-up period, drug treatments and allergen avoidance were standardized. The recurrence of CVA was assessed using the cough visual analogue scale(VAS) score, the total symptom score, the daytime and nighttime symptom score, the children asthma control test(C-TAC) score, and the test for respiratory and asthma control in kids(TRACK) score.

### Diagnostic criteria of CVA

- Cough lasts for more than 4 weeks, and often attacks or worsens during exercise, night and/or early morning, with dry cough as the main cause and no breathing.
- There is no clinical sign of infection, or it is ineffective after long-term antibiotic treatment.
- Anti-asthma drugs are effective in diagnostic treatment.
- Eliminate chronic cough caused by other reasons.
- Positive bronchial provocation test and/or average diurnal variation rate of PEF (continuous monitoring for 2 weeks) ≥  13%.
- Individual or first-and second-degree relatives have a history of atopic diseases, or have positive allergen test.

The above items 1–4 are the basic conditions for diagnosis.

### Exclusion criteria

Children who did not take medicine regularly, who avoided food, who had other basic diseases, and whose family members did not agree to completing the follow-up, were excluded from the study (Fig. 1).

### Mycoplasma pneumoniae

Two groups of children had 2 ml of blood taken from which the serum was separated from the blood and Mycoplasma pneumoniae IgM antibody (MP IgM) testing was carried out, strictly implementing the diagnostic kit for measuring MP antibodies (Passive Particle Agglutinatio) (SERODIA-MYCO II, Fujirebio Inc. Japan). An IgM titer of no less than 1:160 was regarded as positive, indicating an MP infection in children.

### IgE measurement

The serum specific Immunoglobulin E (sIgE) to 14 kinds of common allergens and serum total IgE were detected using the AllergyScreen test (Mediwiss Analytic GmbH, Moers, Germany). The sIgE allergen assays were tested for milk, egg albumin, peanut, cashew nut, walnut, and dust mite; a positive test result was defined as a result of 0.35 kU/L. IgE sensitization was defined as having one or more positive values for serum allergen-specific IgE.

### Treatment

All children with CVA received the standard treatment, including conventional anti-allergy therapy, allergen avoidance, inhaled corticosteroids (ICS), and a long-acting beta-agonist (LABA). Children under six years of age were treated with fluticasone propionate inhalation aerosol (125 μg/PEN), and children over six years of age were treated with Seretide (salmeterol/rocasone inhalation aerosol) (100 μg/PEN) twice daily for three months, with an appropriate dose reduction after three months to once daily depending on the condition. During this period, Vantorin (salbutamol powder spray) (100 ug/PEN) was used when severe coughing occurred for the asthma. Children with the MP infection were treated with Azithromycin (10 mg/kg/d) for four weeks.

### Outcome measures

The primary outcome this study assessed was the date of the CVA diagnosis. All cases were followed-up starting from the index date until whichever occurred first: 12 months later, loss to follow-up, or death.

### Statistical methods

The χ^2^ test was used to determine the baseline distributions of the CHD and non-CHD cohorts. The recurrence rate of CVA was stratified by gender, age group (1–3, 4–6, and 7–14 years), and the follow-up time (≤ 6 months and > 6 months) of both cohorts. The adjusted hazard ratio (aHR) and 95% confidence interval (CI) of the CVA recurrence were determined by multivariate analysis using the Cox proportional hazard regression model. with controls adjusted for CHD classification, MP infection and IgE sensitization. In order to exclude the influence of time-dependent factors, the follow-up time was divided into ≤ 6 months and > 6 months. The aHR of the rate of the CVA recurrence in children with CHD was estimated according to the CHD classification, the MP infection and IgE sensitization. The paired multivariable Cox proportional hazard model was stratified to assess the hazard of the CVA recurrence for both CHD and non-CHD cohorts. All statistical analysis was performed by the SPSS 24.0 software. The log-rank test assessed the differences between the two cohorts by comparing the cumulative incidence curves. GraphPad Prism 8.0 was used for the study’s drawings. A P value of less than 0.05 in a two-tailed test was considered significant.

## Results

We screened 67 CVA children with CHD and 134 children with non-CHD from 2018 to 2019. During the follow-up period, there were four occurrences of censored data in the CHD cohort and seven in the non-CHD group. Table 1 shows a comparison of demographic characteristics and clinical manifestations between the two cohorts. The gender and age distributions of the cohorts were similar. Of the children, 55.2% were male, 40.3% were aged 1–3 years, 28.4% were 4–6 years old and 31.3% were 7–14 years of age. There were no significant differences in family history, laboratory examinations or treatments between the two cohorts at the baseline.

**Table 1:** Baseline characteristics and clinical presentation of 201 children for the CHD and non-CHD cohort

Characteristic	Non-CHD		CHD		*P* value
	N = 134		N = 67		
	N	%	N	%	
*Clinical presentation*					
Age (year)					0.99
1–3	54	40.3	27	40.3	
4–6	38	28.4	19	28.4	
7–14	42	31.3	21	31.3	
Sex					0.99
Male	74	55.2	37	55.2	
Female	60	44.8	30	44.8	
Family history of allergies	123	91.8	57	85.1	0.150
Family history of eczema	64	47.8	30	44.8	0.689
Family history of asthma	26	19.4	14	20.9	0.803
Laboratory examination					
Elevated eosinophil ratio	44	32.8	18	26.8	0.388
Mycoplasma pneumonia positive	69	51.5	38	56.7	0.484
sIgE sensitization	85	63.4	40	59.7	0.607
Food	70	52.2	32	47.8	0.549
Aeroallergen	84	62.7	37	55.2	0.308
Treatment					0.99
Fluticasone Propionate inhalation aerosol (flixotide) + salbutamol	92	68.7	46	68.7	
Salmeterol xinafoate and fluticasone propionate powder for inhalation (seretide) + salbutamol	42	31.3	21	31.3	

*CHD* congenital heart disease

**Figure.2:**
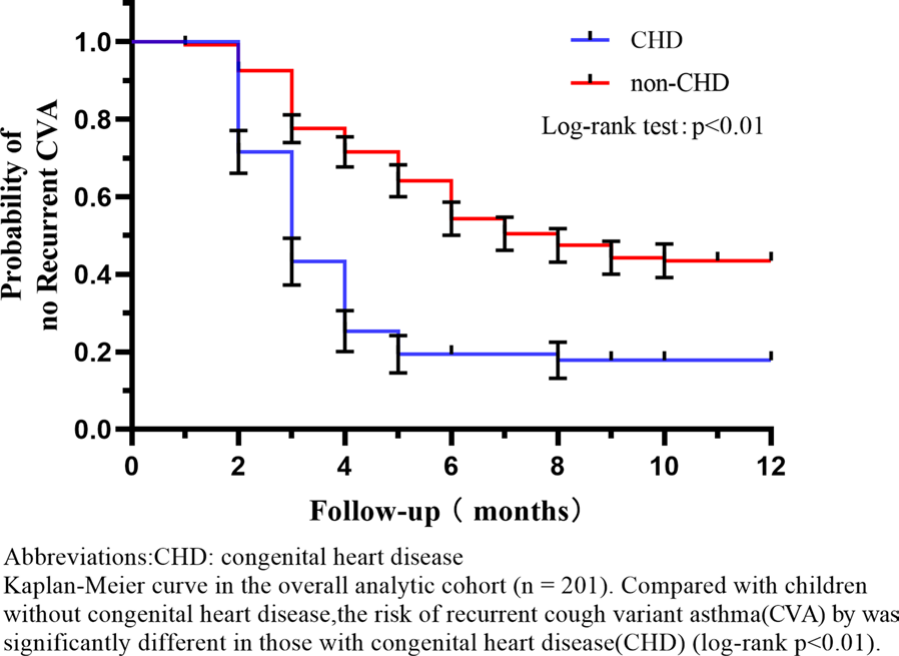
CVA recurrence rate within 12 months of CHD group and non-CHD group

The cumulative hazard of recurrent CVA was significantly higher in the CHD cohort (*P* = 0.001, log-rank test) than in the non-CHD group (see Fig. 2).

Table 2 shows univariate and multivariate analysis of CVA predictors. From the univariate analysis of CVA predictors, CHD classification, MP infection and IgE sensitization were significant differences, while most predictors did not differ between the two virus cohorts. Overall, the multivariable Cox proportional hazards model demonstrated that the recurrence hazard of CVA in the CCHD group was significantly higher than that in the non-CHD cohort (aHR = 3.281; 95% CI 1.648–6.530; *P* = 0.001) after adjusting for CHD classification, MP infection and IgE sensitization; while the recurrence hazard of CVA in the SCHD group was also significantly higher than that in the non-CHD cohort (aHR = 2.555; 95% CI 1.739–3.752; *P* = 0.000). Further, the recurrence hazard of CVA with IgE sensitization (aHR = 2.172; 95% CI 1.482–3.184; *P* = 0.000) had a higher hazard than those without IgE sensitization; the recurrence hazard of CVA with MP infection (aHR = 1.777; 95% CI 1.188–2.657; *P* = 0.005) had a higher hazard than those without the MP infection.

**Table 2:** univariate and multivariate analysis of predictors of CVA

Variables	Univariate analysis		Multivariate analysis	
	HR (95%CI)	*P* value	aHR (95%CI)	*P* value
CHD				
CCHD	3.237 (1.655–6.331)	0.001	3.281 (1.648–6.530)	0.001
SCHD	2.293 (1.575–3.339)	0.000	2.555 (1.739–3.752)	0.000
No	1		1	
Age (year)			–	
1–3	0.985 (0.652–1.488)	0.944	–	
4–6	1.153 (0.739–1.797)	0.531	–	
7–14	1		–	
Male	1.132 (0.800–1.602)	0.484	–	
Family history of allergies	0.731 (0.427–1.253)	0.254	–	
Elevated eosinophil ratio	1.066 (0.736–1.544)	0.734	–	
Mycoplasma pneumonia positive	2.557 (1.770–3.694)	0.000	2.172 (1.482–3.184)	0.000
sIgE sensitization	1.941 (1.324–2.845)	0.001	1.777 (1.188–2.657)	0.005
Standardized treatment	0.875 (0.216–3.539)	0.851	–	

aHR indicates multiple analysis, including CHD classification, MP infection and IgE sensitization

*CHD* congenital heart disease, *CVA* cough variant asthma, *SCHD* simple congenital heart disease, *CCHD* complex congenital heart disease, *sIgE* Serum-specific immunoglobulin E, *HR* hazard ratio, *aHR* adjusted hazard ratio, *MP* mycoplasma pneumoniae

**Figure 3:**
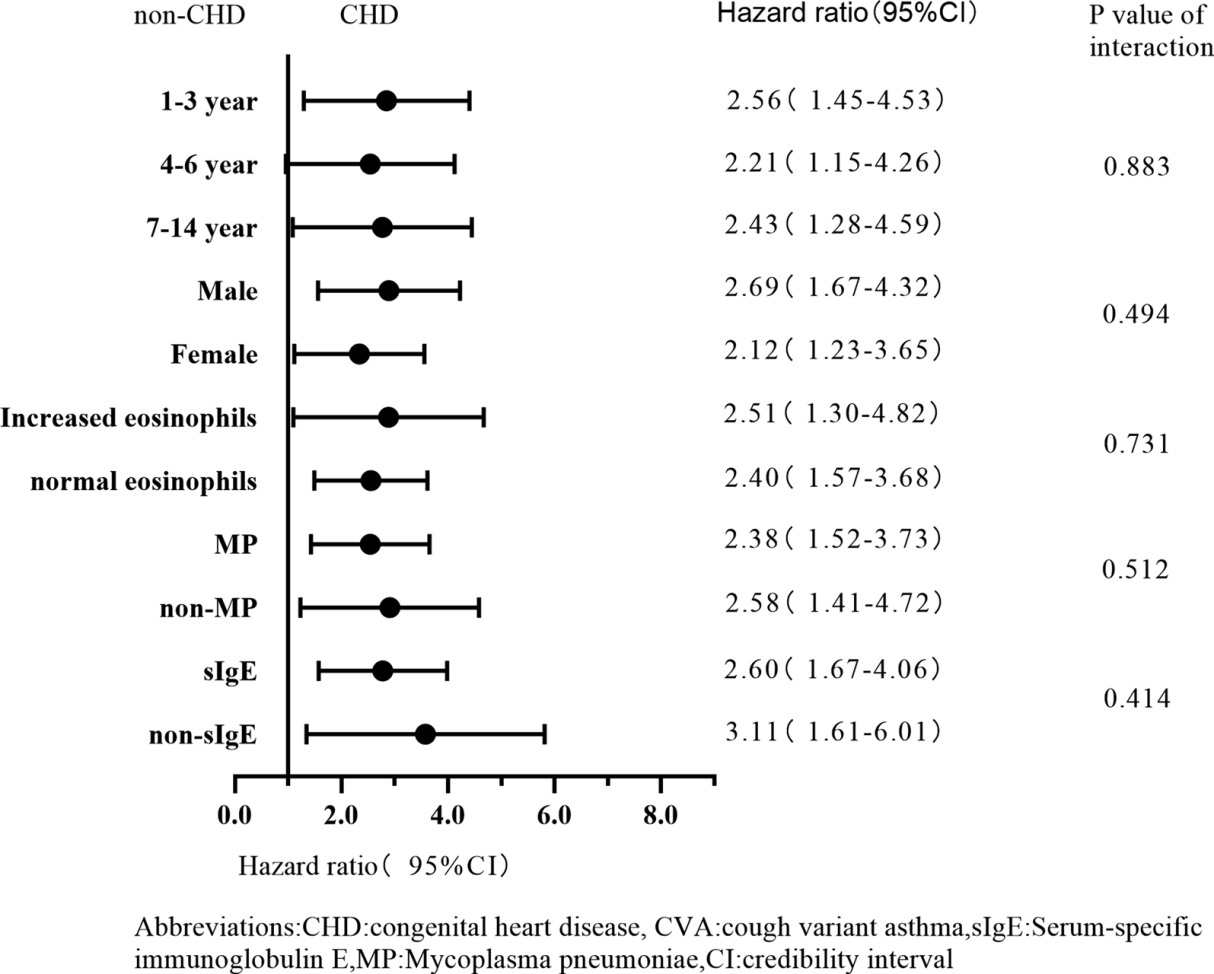
Hazard rations of non-CHD versus CHD cohorts on risks of all-cause mortality in the prespecified subgroups

Figure 3 shows the hazard ratios of non-CHD versus CHD cohorts for risks of all-cause mortality in the prespecified subgroups. The hazard of recurrent CVA was higher in male (HR = 2.69; 95% CI 1.67–4.32; *P* < 0.01) than in female children (HR = 2.12; 95% CI 1.23–3.65; *P* < 0.01). Using age stratification, CVA in CHD patients had the highest recurrent hazard ratio among those aged 1–3 years (HR = 2.56; 95% CI 1.45–4.53; P < 0.01). Children with CHD who had an elevated eosinophil ratio (HR = 2.51; 95% CI 1.30–4.82, *P* < 0.01) had a higher hazard of recurrent CVA than those with a normal eosinophil ratio (HR = 2.40; 95% CI 1.57–3.68; *P* < 0.01), but there was no significant interaction between the hazard ratios of non-CHD versus CHD cohorts for risks of all-cause mortality in relation to the hazard of recurrent CVA (*P* for interaction > 0.05,see Fig. 3).

**Figure 4:**
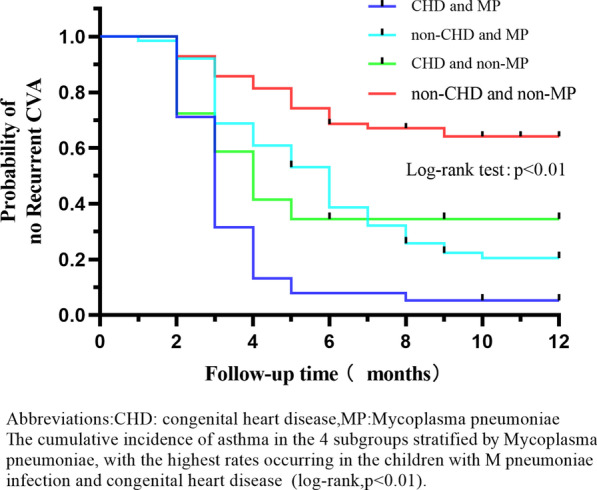
CVA recurrence rate within 12 months of CHD and MP group, non-CHD and MP group, CHD and non-MP group and non-CHD and non-MP group

**Figure 5:**
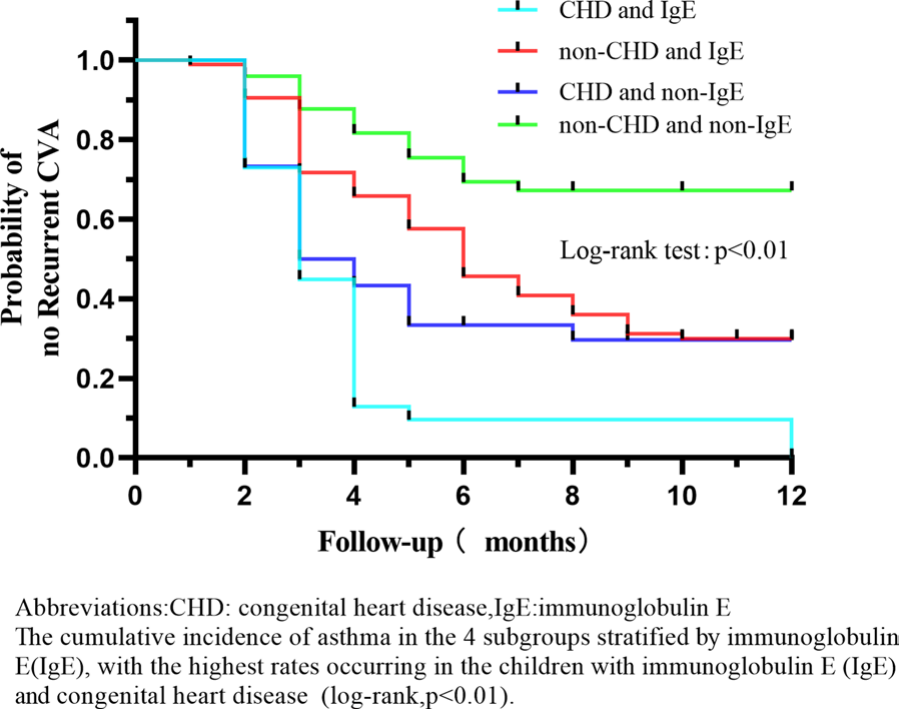
CVA recurrence rate within 12 months of CHD and IgE group, non-CHD and IgE group, CHD and non-IgE group, and non-CHD and non-IgE group

On the one hand, among the four subgroups stratified by MP infection, the children with CHD and MP infection had the highest recurrence rate (*P* = 0.001, log-rank test, see Fig. 4). But there was no significant interaction between CHD and MP infection in relation to the hazard of recurrent CVA (*P* for interaction = 0.512, see Fig. 3). On the other hand, among the four subgroups composed according to IgE sensitization, the hazard of recurrent CVA in children with CHD along with IgE sensitization was highest (*P* = 0.000, log-rank test, see Fig. 5), but there was no significant interaction between CHD and IgE sensitization in relation to the hazard of recurrent CVA (*P* for interaction = 0.414, see Fig. 3).

Table 3 shows that the aHR of these CVA children decreased from 1.852 (95% CI 1.246–2.751; *P* = 0.001) within six months to 0.691 (95% CI 0.088–5.404; *P* = 0.725).

**Table 3:** aHR of CVA recurrence rate stratified by follow-up time between the non-CHD and the CHD cohort

Variables	CHD				Compared with non-CHD	
	No		Yes			
Follow-up time (month)	Event	%	Event	%	aHR (95%CI)	*P* value
≤ 6 month	61/63	96.8	54/55	98.2	1.852 (1.246–2.751)	0.001
> 6 month	14/71	19.7	1/12	8.3	0.691 (0.088- 5.404)	0.725

aHR indicates multiple analysis,including CHD classification, MP infection and IgE sensitization

*CHD* congenital heart disease, *CVA* cough variant asthma, *aHR* adjusted hazard ratio, *MP* mycoplasma pneumoniae

## Discussion

CVA is a particular form of asthma, having a close pathogenesis to typical asthma. Asthma is a disease characterized by infiltration of the mucositis cells in the airway and thickening of the subepithelial basement membrane in the airway [5]. First, we found that those CVA patients with CHD had a higher hazard of recurrence, and that the recurrence hazard for children with CCHD was even higher. We also found that IgE sensitization and MP infection could increase the hazard of CVA recurrence. The effect of CHD on CVA recurrence has hardly been studied previously.

It has been reported that the mechanism for CVA caused by heart disease is bronchial vascular congestion, resulting in bronchial oedema and thickening [6]. In addition, it has been reported that left ventricular dysfunction can lead to abnormal pulmonary function, such as airway hyperresponsiveness or restrictive and obstructive dysfunction [7]. Highly reactive heart failure in patients is thought to be caused by bronchiectasis and submucosal oedema. The incidence of diastolic dysfunction associated with obstruction was over four times higher than that in the control group [8]. This is caused by oxidative stress factors. Although according to current understanding, volume overload in heart disease may also be a cause of inflammation. This additionally leads to airway remodeling [9]. In addition, children with CHD often have bronchial hyperresponsiveness, and this, especially when there is left-sided heart failure, can also damage the lungs. It can lead to pulmonary vascular remodeling, increased pulmonary vascular resistance, and alveolar wall thickening. To some extent, these changes have a protective effect and can prevent pulmonary oedema. Over a long period, however, they may lead to pulmonary hypertension and affect lung function [10].

It is generally believed that CHD is associated with recurrent asthma attacks in children. For example, there is a high prevalence of airway hyperresponsiveness in patients with atrial septal defects (ASD), and it is suggested that airway hyperresponsiveness may be a possible mechanism for recurrent attacks of CVA. Unlike bronchial asthma, 11 asthma-like symptoms associated with ASD are not necessarily experienced every few hours or days, but can be progressive [11]. This better understanding of ASD-associated dyspnea could potentially prevent delays to the treatment of unrepaired ASD. It may also prevent a hazard of complications associated with long-standing right ventricular volume overload and delayed diagnosis of pulmonary function damage after long-term operation. In clinical follow-ups of adult patients with ASD, symptoms and lung function should be monitored for a long time before and after any operations [12]. Therefore, the effect of CHD plays an important role in the recurrence of CVA in children.

In addition, according to the complexity of cardiac malformation, CHD can be divided into SCHD and CCHD. CCHD was found to cause a higher hazard of recurrent CVA than SCHD. Complex blood flow relationships may lead to pulmonary vascular remodeling, an increase in pulmonary vascular resistance and alveolar wall thickening, and pulmonary tracheal remodeling, thus increasing the hazard of CVA recurrence. It has been reported that in some heart defects, especially in the left to right shunt, reversible airway obstruction may be caused by abnormal or dilated vascular anatomical compression due to missing pulmonary valves, vascular rings, or pulmonary slings [13].

The second important finding of this study was that children with IgE sensitization had an increased recurrence rate of CVA. Ciprandi et al. found that allergic children who identified with a positive skin prick test or history of allergic diseases had more frequent and severe respiratory tract infections than non-allergic children [14]. The underlying mechanism for the positive association between IgE sensitivity and pneumonia is still unclear. It may involve a reduction in the Type 1 helper cell (T_h_1) response in IgE sensitized patients, which may be important for the prevention of infections and mucositis, and may also involve a reduced defense against infectious factors associated with general allergic diseases, such as atopic dermatitis [15]. This suggests that IgE sensitization may not have a causal relationship with susceptibility to infection, but that IgE sensitization and infection share a common pathway, or that people who are more susceptible to infection may develop atopic diseases more frequently while maintaining a tendency to infection.

In this study, children with MP infection had a higher hazard of recurrent CVA. Relevant research results abroad have shown that MP infection can induce CVA, and MP is considered one of the pathogenic factors for the acute onset, difficult remission, and deterioration of CVA. However, there is no unified theory on the induction mechanism. Some scholars believe that because MP infection is a specific antigen, it can lead to IgE-mediated airway inflammation and airway hyperreactivity through delayed allergic reaction and immediate allergic reaction or high interference and disorder on T-lymphocyte subsets, and finally lead to CVA [16]. Infants with MP infection, particularly with IgE sensitization during infancy, had the highest hazard [17]. Therefore, MP infection may aggravate the recurrence of CVA.

During the one-year follow-up period, the hazard of recurrent CVA in children with CHD was highest in the first six months, while the aHR decreased after six months. This indicated that time-dependent influencing factors were excluded in this study, and that CHD was a high-hazard factor for CVA recurrence within the first six months.

### Limitations

There are several limitations to this study. First, children younger than one years old were not included in the study. The aim in the future is to study children with CVA from this age group. Second, as this was a single-center study, the result may have been influenced by unpredictable factors. Third, due to the small number of cases, this study could not analyze the impact of each type of CHD on the recurrence of CVA. At the same time, no comparison exists between the influence of pre- and post-operative CHD on CVA recurrence. The number of cases will be increased in future studies to complete the picture.

## Conclusion

In a prospective cohort study of 201 children with CVA, we found a significantly increased hazard of recurrent CVA in children with CHD, especially in CCHD children. In addition, the hazard of recurrent CVA can increase for children with IgE sensitization or MP infection. Clinicians at outpatient clinics need to be aware of this association and thus pay more attention to the hazard of recurrent CVA in children with CHD.

## Data Availability

The datasets used and/or analysed during the current study are available from the corresponding author on reasonable request. All data generated or analysed during this study are included in this published article.

## Abbreviations

CVA: Cough variant asthma
CHD: Congenital heart disease
VAS: Visual analog scale
C-TAC: Children asthma control test
TRACK: Test for respiratory and asthma control in kids
aHR: Adjusted hazard ratio
IgE: Immunoglobulin E
sIgE: Serum-specific IgE
CI: Credibility interval
MP: Mycoplasma pneumoniae
IgM: Immunoglobulin m
ICS: Inhaled corticosteroids
LABA: Long-acting beta agonist
ASD: Atrial septal defect
T_h_1: Type 1 helper cell
GINA: Global initiative on the prevention and treatment of asthma

## References

[1] Boulet L, Reddel HK, Bateman E, Pedersen S, FitzGerald JM, O'Byrne MP (2019). The global initiative for asthma (GINA): 25 years later. Eur Respir J.

[2] Chan A, Aijaz A, Zaidi AN (2020). Surgical outcomes in complex adult congenital heart disease: a brief review. J Thorac Dis.

[3] Rabe KF, Hurst JR, Suissa S (2018). Cardiovascular disease and COPD: dangerous liaisons?. Eur Respir Rev.

[4] Bao YX, Chen AH, Fu Z, Li CC, Liu CH, Li Xiang (2016). Guidelines for the diagnosis and prevention of bronchial asthma in children. Chin. J. Pediatr.

[5] Medina JL, Coalson JJ, Brooks EG, Le Saux CG, Winter VT, Chaparro T (2014). Mycoplasma pneumoniae CARDS toxin exacerbates ovalbumin-induced asthma-like inflammation in BALB/c mice. PLoS ONE.

[6] Faggiano P (1994). Abnormalities of pulmonary function in congestive heart failure. Int J Cardiol.

[7] Jany B, Bals R, Dreher M, Held M, Jany L, Rembert Koczulla A (2019). Expert workshop COPD: lungs and heart—quite often ill together]. Pneumologie.

[8] Folmsbee SS, Gottardi CJ (2017). Cardiomyocytes of the heart and pulmonary veins: novel contributors to asthma?. Am J Respir Cell Mol Biol.

[9] Nishimura Y, Yu Y, Kotani Y, Nishiuma T, Lin S, Maeda H (2001). Bronchial hyperresponsiveness and exhaled nitric oxide in patients with cardiac disease. Respiration.

[10] Dayeh NR, Ledoux J, Dupuis J (2016). Lung capillary stress failure and arteriolar remodelling in pulmonary hypertension associated with left heart disease (group 2 PH). Prog Cardiovasc Dis.

[11] Nassif M, van Steenwijk RP, Hogenhout JM, Lu HL, Bruin-Bon RHACM, Hirsch A (2018). Atrial septal defect in adults is associated with airway hyperresponsiveness. Cong Heart Dis.

[12] Van Riel ACMJ, Blok IM, Zwinderman AH, Wajon EMCJ, Sadee ASJM, Boo MB (2015). Lifetime hazard of pulmonary hypertension for all patients after shunt closure. J Am Coll Cardiol.

[13] Collins LK, Levin TL, Berdon WE, Cowles RA, Newman B (2010). Rudhe syndrome: reversible right middle lobe emphysema in infants with left-to-right shunts—an historical review. Pediatr Radiol.

[14] Ciprandi G, Tosca MA, Fasce L (2006). Allergic children have more numerous and severe respiratory infections than non-allergic children. Pediatr Allergy Immunol.

[15] Rantala A, Jaakkola JJK, Jaakkola MS (2013). Respiratory infections in adults with atopic disease and IgE antibodies to common aeroallergens. PLoS ONE.

[16] Abdel-Warith Abdel-Wahab A, Younis El-Sayed MI, Al-Asgah NA (2016). Potential use of green macroalgae Ulva lactuca as a feed supplement in diets on growth performance, feed utilization and body composition of the African catfish, Clarias gariepinus. Saudi J Biol Sci.

[17] Hasegawa K, Mansbach JM, Bochkov YA, Gern GE, Piedra PA, Bauer CS (2019). Association of rhinovirus C bronchiolitis and immunoglobulin E sensitization during infancy with development of recurrent wheeze. JAMA Pediatr.

